# Evidence for an Invasive Aphid “Superclone”: Extremely Low Genetic Diversity in Oleander Aphid (*Aphis nerii*) Populations in the Southern United States

**DOI:** 10.1371/journal.pone.0017524

**Published:** 2011-03-09

**Authors:** John Scott Harrison, Edward B. Mondor

**Affiliations:** Department of Biology, Georgia Southern University, Statesboro, Georgia, United States of America; University of Arkanas, United States of America

## Abstract

**Background:**

The importance of genetic diversity in successful biological invasions is unclear. In animals, but not necessarily plants, increased genetic diversity is generally associated with successful colonization and establishment of novel habitats. The Oleander aphid, *Aphis nerii*, though native to the Mediterranean region, is an invasive pest species throughout much of the world. Feeding primarily on Oleander (*Nerium oleander*) and Milkweed (*Asclepias* spp.) under natural conditions, these plants are unlikely to support aphid populations year round in the southern US. The objective of this study was to describe the genetic variation within and among US populations of *A. nerii*, during extinction/recolonization events, to better understand the population ecology of this invasive species.

**Methodology/Principal Findings:**

We used five microsatellite markers to assess genetic diversity over a two year period within and among three aphid populations separated by small (100 km) and large (3,700 km) geographic distances on two host plant species. Here we provide evidence for *A. nerii* “superclones”. Genotypic variation was absent in all populations (i.e., each population consisted of a single multilocus genotype (MLG) or “clone”) and the genetic composition of only one population completely changed across years. There was no evidence of sexual reproduction or host races on different plant species.

**Conclusions/Significance:**

*Aphis nerii* is a well established invasive species despite having extremely low genetic diversity. As this aphid appears to be obligatorily asexual, it may share more similarities with clonally reproducing invasive plants, than with other animals. Patterns of temporal and geographic genetic variation, viewed in the context of its population dynamics, have important implications for the management of invasive pests and the evolutionary biology of asexual species.

## Introduction

While studies are few, genetic diversity is believed to facilitate successful biological invasions [1]. In animals, for example higher levels of genetic diversity increased population persistence and colonization success [2] and may contribute to increased range expansion [3], [4]. In invasive plants however, genetic diversity is high in some species, but many successful invaders have little or no genetic diversity (for a review see [5]).

Aphids are valuable study systems for investigating the roles of genetic variation and phenotypic plasticity on population, ecological, and evolutionary dynamics [6], [7], [8]. The Oleander aphid, *Aphis nerii* Boyer de Fonscolombe, is a pest of several plant families including Apocynaceae (*Nerium* and *Vinca*), Asclepiadaceae (*Asclepias*, *Calotropi*, and *Gomphocarpus*), Asteraceae, Convolvulaceae, Euphorbiaceae, and Rutaceae [9]. This aphid, along with its principal host plant (Oleander), is thought to be Mediterranean in origin. *Aphis nerii* has since become a common invasive species in warm temperate and tropical regions of the world [9].

In the United States, the Oleander aphid commonly infests two plant families, Apocynaceae and Asclepiadaceae [10]. Oleander, *Nerium oleander* (Apocynaceae), is a common ornamental plant in southern and coastal states and frequently grows along US highways [11]. Native and ornamental Milkweed (Asclepiadaceae) is the other common US host. Milkweed distribution overlaps with that of Oleander, but extends into northern and central states [12], [13]. Although these two host plant types are ecologically different, both are patchily distributed, contain cardiac glycosides which *A. nerii* sequesters for defense, and are unable to support aphid populations throughout the year [9], [11].

Aphid fitness tradeoffs among host plant species results in selection for host fidelity which can inhibit gene flow and result in the development of host races; i.e., host-associated population genetic structure [7], [14]. Increased use of molecular markers to study aphid populations has revealed that host races are common [15], [16], [17]. There is currently no evidence of differential fitness for *A. nerii* inhabiting species of milkweed that differed in the amount cardiac glycosides that they possess [13]. Fitness tradeoffs in *A. nerii* among different host plant families such as Milkweed and Oleander, to our knowledge, have not been examined.

Aphid species vary in their mode of reproduction from obligate to cyclical parthenogens, but less than 3% of species are strictly clonal [18]. *Aphis nerii* is believed to be an obligate parthenogen; males have never been found in natural populations [9]. Males, and sexual reproduction, have been induced in laboratory lines under short-day conditions [19], [20]. These laboratory-induced sexuals, however, had low fecundity and the extent to which sexual reproduction occurs in nature is unclear [19], [20]. Sexual reproduction and recombination increases variation and sets the stage for selection and adaptation [18], [21]. Conversely, asexual reproduction may limit genetic variation and adaptive potential, but provides reproductive assurance in stable environments and during colonization events [18], [21], [22] (see also [23]).

The genetic structure of aphid populations is shaped spatially and temporally by habitat distribution, dispersal capabilities, and life-cycle. Many aphids, including *A. nerii*, produce winged forms in response to overcrowding and/or decreasing host plant quality [24], [25]. Dispersal range is unknown for most species, but reports of less than one to hundreds of kilometers when habitat is continuous, are not uncommon [26]. *Aphis nerii*, however, is found in patchily distributed habitats throughout the United States, and both Oleander and Milkweed are unable to support aphid populations year round except in the southernmost latitudes of the US [11]. Consequently, most populations are characterized by frequent extinction events followed by re-colonization from unknown source population(s). Spatial and temporal genetic variation is driven by the magnitude of population bottlenecks, the number of founding individuals during re-colonization, and the genetic variation of the source population(s).

The aim of this study was to characterize genetic variation within and among southern US populations of *A. nerii* with the goal of gaining insight into the population dynamics, life history, and ecology of this well-established invasive species. In so doing, we asked the following questions: 1) Is there any evidence of sexual reproduction in US populations of *A. nerii*?; 2) Do aphids inhabiting different hosts comprise host races?; 3) Does the patchy distribution of suitable habitat result in population genetic subdivision over small and/or large geographic ranges?; and 4) Is there temporal variation in population genetic structure? To address these questions, we used microsatellite markers to assess genetic diversity over a two year period within and among three populations separated by roughly 100 km and 3,700 km on two host plant species.

Here, we report no evidence of sexual reproduction or the existence of host races in *A. nerii*. Whilst genetic variation was extremely low within and among populations, the genetic composition of one population was found to change drastically over time. Our findings suggest that *A. nerii* is an efficient colonizer that demonstrates true metapopulation dynamics.

## Methods

### Population Sampling

Aphids were collected from Oleander (*N. oleander*) at two locations in Georgia and one location in central California, June-August 2008, 2009. This “overwintering” period between samples allowed us to assess temporal genetic variation, and determine if sexual reproduction occurred. Sampling locations were: Statesboro, Georgia (SGO; 32°24′N, 81°46′W); Tybee Island, Georgia (TIGO; 31°59′N, 80°50′W); and Concord, California (CCO; 37°57′N, 121°56′W). Sampling areas consisted of multiple patches of Oleander plants in close proximity but intermittently up to 2.5 km apart. To survey genetic diversity within each population, aphids were collected from different parts of the same plant and from as many different plants as possible. To assess genetic variation among aphids inhabiting different host plant species, aphids were collected from Milkweed (*Asclepias amplexicaulis*) at a second site in Statesboro, Georgia (SGM; 32°25′N, 81°47′W) in June 2008.

### Microsatellite analyses

Individual aphids were genotyped at five microsatellite loci (Ago24, Ago66, Ago69, Ago89, and Ago126) using primers originally designed for the Cotton/Melon aphid, *Aphis gossypii* [27]. These loci have been used previously to study at least three different *Aphis* species [27], [28]. No linkage disequilibrium among loci has been detected in any species suggesting that they are unlinked in *A. nerii* as well [27], [28]. These five loci were surveyed in 50 individuals from each population for each year except for SGM where 19 individuals were genotyped (n = 319 total aphids).

DNA was extracted by macerating individual aphids in 70 µl cell-lysis/proteinase-K buffer (10 mM of Tris, 50 mM KCl, 0.5 tween, 0.2 mg/ml proteinase-K, pH. 8.0) followed by incubation for 1 h at 65°C and 15 min at 99°C (Lee and Frost 2002). PCR reactions were carried out in 20 µl final volumes with 2.5 mM MgCl_2_. Reactions consisted of a 4 min denaturation at 94°C, followed by 35 cycles of 30 s at 94°C, 35 s at 58°C, and 45 s at 72°C, and a final 10 min extension step at 72°C.

PCR products were run on small (15 cm×17 cm×0.8 mm), non-denaturing TAE (tris-acetate-EDTA, pH 8.0) buffered polyacrylamide gels of either 9% or 10% concentration, with the lower portion of the gel supplemented with EnhanceIT polymer (Elchrom Scientific, Switzerland). Gels were run for 2 h at 30 mA, stained with ethidium bromide, and visualized on a UV light box. Allele sizes at each locus were estimated using the M3 size standard (Elchrom Scientific, Switzerland).

Observed and expected heterozygosities were calculated for each locus in each population. Deviations from Hardy-Weinberg expectations were tested according to Guo and Thompson [29] and estimates of the inbreeding coefficient *F*_IS_ [30] were estimated using FSTAT v. 2.9.3 [31]. Molecular subdivision among populations and over time were calculated by estimating both *F*_ST_ and *R*_ST_ using the program FSTAT v. 2.9.3 [31]. The significance of *F*-statistics was tested using the randomization procedure available in FSTAT using 5000 permutations. Estimates of genetic distance between multilocus genotypes (MLGs, see below) (δμ^2^ and D_SW_) were calculated according to Shriver et al. [32]. A genotypic diversity index was calculated as the ratio of the number of distinct genotypes out of the total number of samples (G/N ratio). We also compared the ratio of observed multilocus genotypic diversity (G_O_) to that expected under conditions of sexual reproduction (G_E_), as described by Stoddart and Taylor [33]. The presence of null alleles in the genotype data was estimated using Microchecker [34].

## Results

All five microsatellite loci used in this study were polymorphic in *A. nerii*. The number of alleles was low; 3 to 4 alleles per locus. Three of the five loci each had an observed heterozygosity (H_O_) of 0.143, the two loci an H_O_ of 1. Null alleles were not detected for any loci in any populations sampled.

Extremely low levels of genotypic variation were observed in *Aphis nerii*. Only 2 multi-locus genotypes (MLGs) or “clones” were detected among the 319 individuals assayed across all populations and both years (Table 1). MLG 1 was dominant both spatially and temporally, comprising 84.3% of the samples. The remaining 15.7% of the samples consisted of MLG 2. MLG 2 was found in one population (TIGO) in 2009 only. The MLG's differed in levels of heterozygosity, with 2 of 5 loci (40%) heterozygous in MLG 1 and 5 of 5 (100%) in MLG 2.

**Table 1:** Multi-locus genotypes among genotypes and across years.

	Year		2008				2009	
Locus	Population^1^	SGO(n = 50)	SGM(n = 19)	TIGO(n = 50)	CCO(n = 50)	SGO(n = 50)	TIGO(n = 50)	CCO(n = 50)
Ago24		140/148^2^	140/148	140/148	140/148	140/148	134/138	140/148
Ago66		160/160	160/160	160/160	160/160	160/160	156/166	160/160
Ago69		100/100	100/100	100/100	100/100	100/100	90/94	100/100
Ago89		171/171	171/171	171/171	171/171	171/171	155/161	171/171
Ago126		169/175	169/175	169/175	169/175	169/175	171/179	169/175
“Clone” designation		Clone 1	Clone 1	Clone 1	Clone 1	Clone 1	Clone 2	Clone 1

1 SGO = Statesboro, Georgia - Oleander; SGM = Statesboro, Georgia - Milkweed; TIGO = Tybee Island, Georgia - Oleander; CCO = Concord, California – Oleander.

2 Numbers indicate estimated allele sizes for each locus.

Despite the low level of genotypic diversity observed, the genotypes of the two MLGs were divergent. That is, the two MLGs did not share any alleles, clearly indicating that they were not the product of recombination through sexual reproduction. Estimates of genetic distance, based on the stepwise mutation model, were large (δμ^2^ = 15.35; D_SW_ = 15.35) suggesting that the MLGs do not share a close genealogical relationship [35].

No evidence of sexual or mitotic recombination was found. First, no homozygous allelic arrangements were found at any of the heterozygous loci (Table 1). Second, levels of heterozygosity were high, ranging from 0.40 to 1.00 (Table 2), and there were significant deviations from Hardy-Weinberg expectations at polymorphic loci within all populations due to heterozygote excess. Third, the ratio of the number of observed genotypes (G) to the number of individuals sampled (N) ranged from 0.02 to 0.05 (0.024±0.011, mean±SD), and the ratio of the observed multi-locus genotypic diversity to that expected under sexual reproduction (G_O_/G_E_) from 0.141 to 0.048 (0.128±0.035, mean ± SD). Fourth, estimates of F_IS_ were −1.0 for all populations.

**Table 2:** Comparison of genetic parameters among populations and across years.

	Year		2008				2009	
	Population^1^	SGO(n = 50)	SGM(n = 19)	TIGO(n = 50)	CCO(n = 50)	SGO(n = 50)	TIGO(n = 50)	CCO(n = 50)
Number of MLGs		1	1	1	1	1	1	1
“Clone” Designation		1	1	1	1	1	2	1
Mean # alleles/locus		1.400	1.400	1.400	1.400	1.400	2.000	1.400
G_O_/G_E_		0.141	0.141	0.141	0.141	0.141	0.048	0.141
G/N		0.020	0.050	0.020	0.020	0.020	0.020	0.020
H_O_		0.400	0.400	0.400	0.400	0.400	1.000	0.400
H_E_		0.202	0.205	0.202	0.202	0.202	0.505	0.202
F_IS_ (multi-locus)		−1.000	−1.000	−1.000	−1.000	−1.000	−1.000	−1.000

1 SGO = Statesboro, Georgia - Oleander; SGM = Statesboro, Georgia - Milkweed; TIGO = Tybee Island, Georgia - Oleander; CCO = Concord, California - Oleander.

To test for a non-random distribution of genetic variation among host plant species, we sampled *A. nerii* from two common host plants (Oleander and Milkweed) from Statesboro, GA (SGO and SGM, respectively). All samples from both hosts consisted of MLG 1, indicating that there is no host associated subdivision at this location (*F*_ST_ = 0, *R*_ST_ = 0) (Table 3).

**Table 3:** Pairwise molecular subdivision *F*_ST_ (below diagonal) and *R*_ST_ (above diagonal) between populations and across years.

Year			2008				2009	
	Population^1^	SGO	SGM	TIGO	CCO	SGO	TIGO	CCO
	SGO	-	0.000	0.000	0.000	0.000	**0.787**	0.000
2008	SGM	0.000	-	0.000	0.000	0.000	**0.758**	0.000
	TIGO	0.000	0.000	-	0.000	0.000	**0.787**	0.000
	CCO	0.000	0.000	0.000	-	0.000	**0.787**	0.000
	SGO	0.000	0.000	0.000	0.000	-	**0.787**	0.000
2009	TIGO	**0.650**	**0.609**	**0.650**	**0.650**	**0.650**	-	**0.787**
	CCO	0.000	0.000	0.000	0.000	0.000	**0.650**	-

1 SGO = Statesboro, Georgia - Oleander; SGM = Statesboro, Georgia - Milkweed; TIGO = Tybee Island, Georgia - Oleander; CCO = Concord, California – Oleander.

**Bold values** indicate p<0.0005.

There was no genotypic variation within any population for either 2008 or 2009, suggesting that each population was composed of a single genotypic “clone” (Table 1). The geographic distribution of MLGs differed between years. Samples collected in 2008 from Tybee Island, Georgia; Statesboro, Georgia; and Concord, California comprised a single MLG, indicating no population subdivision (*F*_ST_ = 0, *R*_ST_ = 0) (Table 3). The Tybee Island, Georgia samples differed significantly from both the Statesboro, Georgia and Concord, California samples in 2009 (*F*_ST_ = 0.650, *R*_ST_ = 0.787) (Table 3). During the 2009 sampling period, the Tybee Island population consisted solely of MLG 2 individuals which were not found in any other population. Between 2008 and 2009 there was, interestingly, a complete change in the genetic composition of the Tybee Island population from MLG 1 to MLG 2.

## Discussion

Small, genetically-uniform populations are subject to ecological and evolutionary forces (i.e., genetic bottlenecks and genetic drift) which threaten population persistence, in both native and introduced habitats [1]. In animals, higher genetic diversity is often associated with an increased ability to establish viable populations in novel environments [1]. This is not necessarily true for plants, however; some species are very successful with little or no genetic diversity, particularly those that are clonally-reproducing, self-pollinating, or apomictic [5]. Here, we analyzed the genetic patterning of the Oleander aphid, *A. nerii* to better understand the ecology, life history, and population dynamics of this well established invasive species. We found that Oleander aphids are remarkably invasive throughout the southern United States, with extremely low genetic diversity.

### Reproduction and Life History

*Aphis nerii* is believed to be obligately parthenogenetic, based on the complete absence of males under natural conditions [9]. Laboratory lines of the aphid derived from populations in Kyoto, Japan produced males when exposed to short-day conditions [19], [20], suggesting that the ability to sexually reproduce is retained in at least some asexual lineages from some populations. From genotypic data, we found no evidence for sexual reproduction in any of the populations we examined, although, of course, further sampling may yet reveal sexual forms. Obligate parthenogenesis is supported by the lack of expected recombinant genotypes observed over the two year sampling period. A high level of heterozygosity between genotypes is consistent with expected genotypic patterns for long term asexual populations; i.e., the “Meselson effect” [36]. That is, in long term asexual populations, heterozygosity is expected to increase because allelic pairs within a genome will continue to diverge over time while meiotic recombination and segregation do not occur to mix and purge alleles [37], [38], [39]. This genotypic pattern has been observed in several primarily parthenogenetic taxa [40], [41], [42], [43], [44]. Our results are consistent with this pattern, suggesting that sexual reproduction is rare or non-existent in natural populations of *A. nerii* in the southern US.

### Geographic Genetic Variation

The most striking pattern observed in this study was the low level of genotypic diversity within and among populations over a large geographic area. We observed only two MLGs and a maximum estimate of genotypic diversity (G/N) within any population of 0.05. Several similar studies of other aphid species using comparable numbers of loci (4 to 7) have revealed higher levels of genetic variation than that observed in *A. nerii*. For example, long term asexual populations of the pea aphid, *Acyrthosiphon pisum* (Harris) in Japan were composed of five to seven MLGs, and cyclically parthenogenetic populations harbored much greater diversity than did asexual populations [45]. Genotypic diversity (G/N) estimates in this species range from 0.10 to 0.69 [45]. Several other aphid species show similar or greater levels of genotypic diversity as *A. pisum*, both within and among populations [25], [46], [47], [48], [49].

Aphid populations are sometimes composed of a small number of dominant genotypes (“clones”) and many low frequency (rare) genotypes [42], [49], [50], [51], [52]. The term “superclone” has been used to describe genotypes that comprise 40–60% of a population in a region [53]. For example, Peccoud *et al.* [52] sampled recently introduced asexual populations of *A. pisum* in Chile. Among the 432 individuals sampled over a 570 km range, 16 MLG's were identified with three MLG's comprising≈90% (47%, 29%, and 14%) of the diversity. Compared to previous studies, the pattern we observe in *A. nerii* is extreme even for “superclones”. Within any sampling year, each population consisted of a single MLG. In 2008, all populations sampled from both California and Georgia showed only a single MLG (MLG 1). In 2009, populations from Statesboro, Georgia and California comprised the same MLG (MLG 1), whilst a population from Tybee Island, Georgia comprised a single but entirely different MLG (MLG 2).

Variation of morphometric and life history traits in *A. nerii* has been assessed within and among populations from California, Iowa, and Puerto Rico [12]. Significant variation was observed within populations for traits such as maturation time, fecundity, and wing length while only the proportion of winged offspring differed among populations [12]. Assuming low genetic diversity in this population, these data suggest that phenotypic plasticity and maternal effects may be of utmost importance in shaping the population dynamics of this parthenogenetic species [23], [54], [55].

The genetic uniformity observed in *A. nerii* likely results from successive genetic bottlenecks or founder events. Subsequent clonal propagation and rapid spread of genotypes throughout the host range would be followed by rapid clonal competition/selection resulting in a few geographically-widespread and dominant clones (but see also [56]). These processes would occur during the initial introduction of this species into the US and/or during annual extinction and recolonization events. A study of *A. nerii* on *N. oleander* in California clearly showed annual colonization and extinction cycles [11], corresponding with our own observations. Patches of host plants are typically colonized by aphids in late Spring (May or June) followed by rapid increases in population sizes which peak in mid-summer. Aphid numbers decline rapidly in early Fall (September or October), resulting in population extinction [11]. In the southernmost US latitudes, *A. nerii* has the potential to overwinter as adults, as freezing temperatures are uncommon. Host plant quality, however, substantially decreases in Fall and Winter likely driving the population decline [11].

Milkweed (*Asclepias* spp.) is the most common host of *A. nerii* in the northern US where *N. oleander* grows infrequently [9]. *Asclepias* species are perennial plants that sprout in Spring, bloom in early Summer, and then set seed and die-back in the Fall. The life history of this host plant would require *A.nerii* to have a secondary host (though this has not previously been reported) or to produce sexuals to produce overwintering eggs (and as previously noted, has only been noted under laboratory conditions). On both Oleander and Milkweed, a regular pattern of colonization, rapid increase, and population extinction would be expected.

### Host Associated Genetic Variation

Groeters [13] found no evidence of fitness trade-offs in *A. nerii* when feeding on different species of milkweed, but differences between Oleander and Milkweeds were not examined. Prior to this study, it was not known if *A. nerii* populations inhabiting Oleander and Milkweed were genetically different. Several aphid species have “host races” or show non-random distribution of genetic variation among host plants, including the Pea aphid [14], [52], the Grain aphid, *Sitobion avenae* (F.) [54], [57], the Cotton /Melon aphid, *Aphis gossypii* Glover [15] and others [16], [17], [58]. This phenomenon undoubtedly results from habitat choice/host fidelity inhibiting interpopulation gene flow [14], [59]. If the same process of habitat choice/host fidelity applied to *A. nerii* populations, we would have expected to see genetic variation between populations inhabiting Oleander and those inhabiting Milkweed, regardless of geographic proximity. Our findings suggest that there is no selection for host specificity and that *A. nerii* is a polyphagous, i.e. generalist species, although more data are required to confirm this contention. This pattern is consistent with Lynch [60] who suggested that obligate parthenogenetic species evolve to be ecological generalists (selection favors clones that can survive in all environments).

In summary, populations of *A. nerii* surveyed in this study show the genetic signature of obligate parthenogenetic reproduction, supporting previous reports that the species is indeed obligately asexual. Within any sampling period, each population was composed of a single MLG. This level of variation is remarkably low compared to the variation observed in other aphid species presumed to be largely or completely asexual [42], [49], [52]. Furthermore, only two MLGs were identified among all populations, and populations separated by as far as 3,600 km were genetically homogenous. Temporal variation occurred in one population, one MLG was completely replaced by another between years. There was no correlation between host plant and MLG. In an ecological context, our results suggest that *A. nerii* is a generalist species with strong dispersal capabilities. As patches of host plants are colonized by few individuals that reproduce rapidly through parthenogenesis, founder individuals likely come from a source population(s) characterized by low genetic diversity.

Despite having extremely low genetic diversity Aphis nerii is a well established invasive species. Understanding the temporal and geographic genetic variation of *A. nerii* provides great insight for the management of invasive pests and the population dynamics of clonal organisms.
